# Yeast expressed recombinant Hemagglutinin protein of Novel H1N1 elicits neutralising antibodies in rabbits and mice

**DOI:** 10.1186/1743-422X-8-524

**Published:** 2011-11-29

**Authors:** TN Athmaram, Shweta Saraswat, SR Santhosh, Anil Kumar Singh, VVS Suryanarayana, Raj Priya, N Gopalan, Manmohan Parida, PV Lakshmana Rao, R Vijayaraghavan

**Affiliations:** 1 Division of Virology, Defence Research and Development Establishment, Ministry of Defence (Govt. of India), Gwalior, MP-474 002, India; 2 Institute of Aerospace Medicine, Indian Air force, Airport Road, Vimanapura PO Bangalore, Karnataka, India; 3 Molecular Virology Laboratory, Indian Veterinary Research Institute, Hebbal, Bangalore-560024, India; 4 Bioprocess and Scale up Facility, Defence Research and Development Establishment, Ministry of Defence (Govt. of India), Gwalior, MP-474 002, India; 5 Defence Research and Development Establishment, Ministry of Defence (Govt. of India), Gwalior, MP-474 002, India

**Keywords:** Hemagglutinin, H1N1, *Pichia pastoris*, secreted expression, Influenza recombinant vaccine

## Abstract

Currently available vaccines for the pandemic Influenza A (H1N1) 2009 produced in chicken eggs have serious impediments *viz *limited availability, risk of allergic reactions and the possible selection of sub-populations differing from the naturally occurring virus, whereas the cell culture derived vaccines are time consuming and may not meet the demands of rapid global vaccination required to combat the present/future pandemic. Hemagglutinin (HA) based subunit vaccine for H1N1 requires the HA protein in glycosylated form, which is impossible with the commonly used bacterial expression platform. Additionally, bacterial derived protein requires extensive purification and refolding steps for vaccine applications. For these reasons an alternative heterologous system for rapid, easy and economical production of Hemagglutinin protein in its glycosylated form is required. The HA gene of novel H1N1 A/California/04/2009 was engineered for expression in *Pichia pastoris *as a soluble secreted protein. The full length HA- synthetic gene having α-secretory tag was integrated into *P. pastoris *genome through homologous recombination. The resultant Pichia clones having multiple copy integrants of the transgene expressed full length HA protein in the culture supernatant. The Recombinant yeast derived H1N1 HA protein elicited neutralising antibodies both in mice and rabbits. The sera from immunised animals also exhibited Hemagglutination Inhibition (HI) activity. Considering the safety, reliability and also economic potential of Pichia expression platform, our preliminary data indicates the feasibility of using this system as an alternative for large-scale production of recombinant influenza HA protein in the face of influenza pandemic threat.

## Background

Influenza viruses belonging to the *Orthomyxoviridae *family are enveloped viruses with segmented negative sense RNA genome surrounded by a helical symmetry shell. The 2009 H1N1 novel virus derived its genes from viruses circulating in the pig population [1-3]. Current influenza vaccines protect against homologous viruses but are less effective against antigenic variants and provide little protection against a different subtype. In the event of a pandemic, existing vaccines may be ineffective because the manufacturing process requires at least six months from identification of the pandemic strain to distribution which is insufficient time to prevent wide-scale morbidity or mortality. New vaccine strategies are therefore needed that can both accelerate production and provide broader spectrum protection. In case of Influenza virus, it is the HA surface glycoprotein that mediates virus entry and is the most important target of antibody-mediated protection [4]. Cellular proteases cleave the HA precursor (HA0) into HA1 and HA2 subunits. The HA1 surface subunit mediates the binding to cell surface sialic acid receptors and the HA2 transmembrane subunit mediates membrane fusion between viral and endosomal membranes after endocytosis [5]. Both during infection and vaccination, HA protein is known to elicit neutralizing antibodies. From the HA antigenic maps, it is evident that HA1 is the major target of neutralizing antibodies that inhibit virus binding to target cells and are classically detected by the hemagglutination inhibition (HI) assay [6-8]. Hence recombinant HA protein based subunit vaccines offer an alternative over conventional vaccine strategies that could save several months of manufacturing time, since the HA gene of the newly circulating strain is available shortly after virus isolation or nucleotide sequencing of HA gene. In contrast to conventional approaches there is no need for live influenza virus or large quantities of eggs, and subunit vaccines could be deployed earlier in the pandemic for effective reduction of morbidity and mortality. It is also economical to produce these vaccines capable of inducing antibody that can neutralize the circulating strain of influenza. As it is very important to produce the antigenic protein in its native soluble and glycosylated form, prokaryotic system like bacteria may not be suitable for making this vaccine protein. *E.coli *being prokaryote is unable to correctly fold the foreign protein and perform other post-translational modifications thus limiting the types of protein(s) that can be expressed. Since the protein product may be typically obtained as insoluble, mis-folded inclusion bodies, subsequent solubilization and re-folding steps are required [9,10]. This incorrect folding can be a result of inadequate intracellular chaperone concentrations or the reducing environment of the cytoplasm [11]. *E. coli *is therefore not generally suitable for use in expression studies with proteins that contain a high level of disulphide connectivity or proteins that require other types of post-translational modifications such as glycosylation [12,13]. *E.coli *expressed proteins also tend to retain their amino-terminal methionine, which may affect protein stability as reported earlier [14,15].

Previous studies on bacterially expressed HA proteins of H5N1 avian influenza virus (AIV) have reported that in the absence of glycosylation, the newly synthesized HA proteins are not likely to fold properly or trimerize like native HA molecules, and may not present native conformational epitopes, which are important for generation of an effective protective immune response [16-18]. Indeed majority of the previous studies did not demonstrate proper folding and/or oligomerization of the HA proteins produced in prokaryotic systems [16-20]. The recombinant protein expressed in *E.coli *as inclusion bodies, requires careful optimization of the re-folding conditions [21-23]. Optimization of such re-folding conditions may be difficult to achieve and is also time consuming [24-26]. In addition, this would also result in significant losses of the recombinant protein, lower productivities and increased costs of manufacture of the expressed protein. Expression of HA in insect cells and mammalian cells are under development and/or clinical trials [27-29]. The main challenge to the recombinant technology is to ensure that the HA products resemble the native virion-associated trimeric spike proteins and can elicit robust immune responses targeting protective conformational epitopes of HA.

Yeast (*Pichia pastoris*) has emerged as an ideal organism to express viral antigens because yeast glycosylate proteins more similarly to mammals than bacteria. Compared with insect or mammalian cells, expression of recombinant proteins in yeast could present a viable alternative in terms of large scale vaccine production and a short time line suitable for rapid response in influenza pandemic. *Pichia pastoris *is methylotrophic yeast, capable of metabolizing methanol as its sole carbon source. It can metabolise methanol using the enzyme alcohol oxidase during oxidation that take place in peroxisomes. The formaldehyde and hydrogen peroxide formed are sequestered within the peroxisomes. Alcohol oxidase has a poor affinity for oxygen and *Pichia pastoris *compensates by generating large amounts of the enzyme. Hence the promoter regulating the production of alcohol oxidase is widely used to drive heterologous protein expression in *Pichia*. Multiple copy integration of recombinant genes in *Pichia *has been demonstrated to increase expression of the desired protein in many cases [30-36]. More recently, *P. pastoris *has been used to express therapeutic proteins that have entered clinical trials [37]. Further development in the field of therapeutic glycoprotein production with *P.pastoris *strains is expected due to recent advances in genetic engineering of human glycosylation pathways into yeasts [38-40]. With the ability to replicate certain human glycosylation patterns, yeast-based expression platforms offer an attractive alternative to current mammalian or insect cell culture processes due to a variety of additional advantages *viz *cheaper operating costs, simple chemically defined media and no viral contamination. In light of the above facts, we have investigated the feasibility of using *Pichia pastoris *as an expression host for recombinant production of H1N1 HA protein and elicitation of neutralising antibodies against the same protein in BALB/c mice and rabbits.

## Results

### Generation of recombinant *Pichia pastoris *with multi copy integrants of H1N1 HA gene

**Figure 1:**
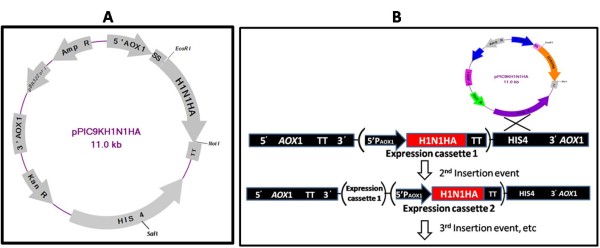
**Vector map of the recombinant construct and Schematic diagram of genetic recombination in Pichia**. Panel 1A: Plasmid map of recombinant yeast transfer vector pPIC9KH1N1HA. Panel 1B: Schematic diagram showing the genetic recombination event that result in the formation of Pichia transformant with multiple-copy integrants(The gene of interest (H1N1HA) is positioned between *Eco *RI and *Not *I sites under the control of AOX1 promoter, the secretory signal (SS), transcription termination (TT) signal sequences are on 5' and 3'end of the H1N1HA gene respectively. HIS 4 locus is carrying the *Sal *I recognition sequence and the integration of the transgene within the Pichia genome will be at His 4 locus).

Full length H1N1 HA synthetic gene of A/California/04/2009 was inserted at *Eco*RI -*Not *I sites into pPICK9K yeast transfer vector under AOX1 promoter in fusion with *S.cervecea *alpha secretory signal at N-terminus. The resultant pPICK9KH1N1HA (Figure 1A), linearized with *Sal *I restriction enzyme after transformation in *P.pastoris via *electroporation yielded 365 His+ transformants per 10μg of DNA used. Further selection of all transformants on Geneticin containing YPD plates resulted in 145 colonies showing resistance to different concentrations of Geneticin tested. Eighty one colonies appeared on the plate containing 250μg/ml Geneticin. Whereas the selection plates with 500μg/ml and 750μg/ml Geneticin had 43 and 21 colonies respectively. Genomic DNA PCR from twenty selected yeast transformants using AOX 1 forward and HA gene specific reverse primer resulted in amplification of approximately 1.96 kb products as expected. Fourteen out of twenty DNA samples amplified the expected 1.96 kb DNA along with known positive control, whereas, DNA from non recombinants did not show any amplification (Data not shown). Multiple copy integrants having more than four copies of HA gene were selected based on antibiotic sensitivity assay and Genomic DNA PCR. The clones that were resistant to higher concentrations of Geneticin also showed intensive bands in genomic DNA PCR and these results were in agreement with that of Geneticin sensitivity assay.

### Optimisation of expression parameters and purification of soluble glycosylated HA0 protein from yeast culture supernatant

**Figure 2:**
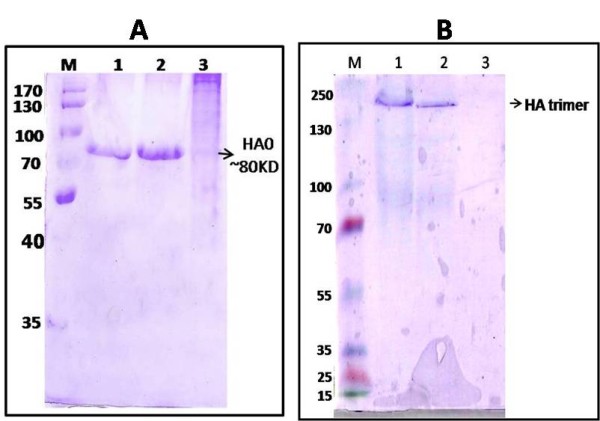
**PAGE analysis of the culture supernatants under denaturing and native conditions demonstrating the secreted expression of recombinant HA protein as HA monomers and HA trimers respectively**. Panel 2A: Methanol induced culture supernatants under denaturing conditionLane M: Pre-stained Protein marker (Fermentas, USA, #SM 0671) Lane 1: Cell culture supernatant from Pichia multiple copy integrant Clone A Lane 2: Cell culture supernatant from Pichia multiple copy integrant Clone B Lane 3: Cell culture supernatant from Negative clone Panel 2B: Methanol induced culture supernatants under native conditionLane M: Pre-stained Protein marker (Fermentas, USA, #SM 1811) Lane 1: Cell culture supernatant from Pichia multiple copy integrant Clone B Lane 2: Cell culture supernatant from Pichia multiple copy integrant Clone A Lane 3: Cell culture supernatant from Negative clone.

We have employed *S. cerevisiae *α-mating factor pre-pro leader sequence secretory signal (SS) upstream to the HA gene in the pPICK9KH1N1HA construct. This signal sequence comprises a 19 amino acid signal peptide (pre-sequence), followed by a 60 amino acid pro-region. The endopeptidase and kex2 protease of Pichia cleaves the Pre and Pro fusion fragments of the expressed protein respectively, resulting in the release of the matured, fully processed HA protein. All the fourteen His+ Mut+ colonies that were found positive through PCR were selected for inducing the expression of the target gene. Out of fourteen clones induced, two clones showed better expression of the HA recombinant protein of size ~80 KDa after 46 hr induction. Whereas no specific protein bands were detected in pPICK9K vector transformed yeast and un-induced positive transformants in this region. Studies conducted to scale-up the expression level with different induction period and methanol concentrations revealed that 46 hr of post-induction with 2% methanol concentration is optimum for better expression of H1N1 HA protein in shaker flask culture level (Data not shown). Upon quantification, the concentration of the HA protein expressed was found to be 60 mg/Lt of the culture supernatant. However, the level of expression could not be further elevated above this scale with either increased methanol concentration or increased duration of incubation. With the above optimised expression conditions, properly folded glycosylated HA0 was secreted into the medium as soluble protein. The expressed HA0 protein ran as a single band on SDS-PAGE with the anticipated molecular weight of approximately 80 KDa (Figure 2A). No significant trimers or dimeric forms of the HA protein were detected on the gel under denaturing conditions. However, high molecular weight protein corresponding to the trimeric form of HA (~240 KDa) was noticed along with HA0 monomers under native conditions (Figure 2B).

**Figure 3:**
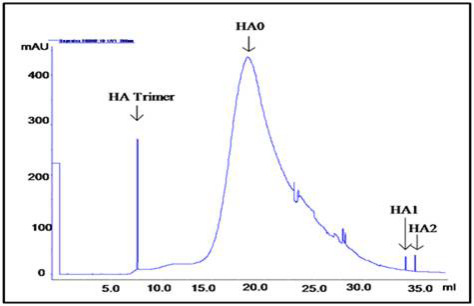
**FPLC purification of yeast derived HA protein *via *size exclusion chromatography**.

FPLC size exclusion chromatography purification of the expressed native protein revealed a broad major peak corresponding to a known protein of molecular weight of ~80 KDa that correlated with that of the intact HA0 protein. The peaks corresponding to the HA timers also formed a narrow intense peak that was further confirmed *via *immunoblotting (Figure 3). Minor peaks corresponding to the breakdown products of HA0 into HA1/HA2 were also observed. However the concentration of these breakdown products were significantly very less in comparison to the intact HA0 as also seen on the native Coomassie stained gel (Figure 2B).

### Western blot analysis of the yeast expressed protein

**Figure 4:**
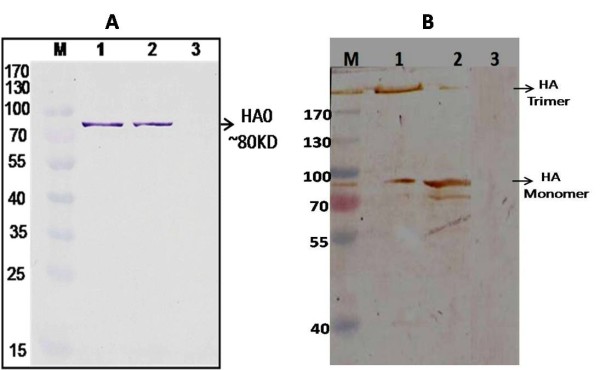
**Western Blot analysis of the culture using H1N1 HA specific antibodies confirming the secreted expression of recombinant HA protein supernatants under denaturing and native conditions**. Panel 4A: Under denaturing condition: Lane M: Pre-stained Protein marker (Fermentas, USA, #SM 0671) Lane 1: Cell culture supernatant from Pichia multiple copy integrant Clone A Lane 2: Cell culture supernatant from Pichia multiple copy integrant Clone B Lane 3: Cell culture supernatant from negative clone Panel 4B: Under native condition: Lane M: Pre-stained Protein marker (Fermentas, USA, #SM 0671) Lane 1: Cell culture supernatant from Pichia multiple copy integrant Clone A Lane 2: Cell culture supernatant from Pichia multiple copy integrant Clone B Lane 3: Cell culture supernatant from negative clone.

The recombinant protein obtained from positive Pichia transformants after methanol induction were separated on PAGE gel under both denaturing and native conditions were subsequently transferred onto PVDF membranes. Western blotting using H1N1 HA specific polyclonal antibodies confirmed the authenticity of the expressed proteins. Appropriate positive signals were obtained only in case of the culture supernatants of positive yeast transformants, whereas the protein sample transferred from Pichia transformed with pPICK9K negative control did not develop any signal on the membrane. HA0 monomers and HA trimers were recognised by the H1N1 HA specific antibodies used in the present study both under denaturing and native conditions respectively (Figure 4A and 4B).

### Trypsin cleavage analysis of yeast derived H1N1 HA0 recombinant protein

**Figure 5:**
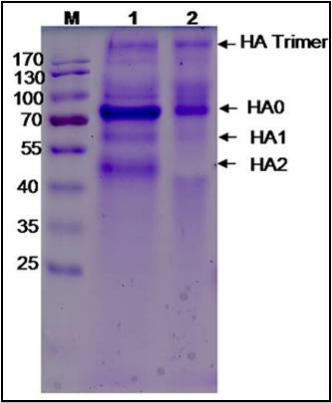
**Native PAGE analysis of the yeast derived H1N1HA digested with trypsin **Lane M: Pre-stained Protein marker (Fermentas, USA, #SM 0671) Lane 1: Recombinant H1N1HA after digestion with trypsin Lane 2: Negative control (Recombinant H1N1HA mock digested).

Upon trypsin digestion, the expressed HA0 showed cleavage into HA1 (55-60 KD) and HA2 (40-45 KD) as seen on native PAGE (Figure 5, lane 1). Whereas in case of mock digested HA protein, no such fragments were noticed (Figure 5, lane 2).

### Immunization of mice and rabbits with yeast derived H1N1HA recombinant protein

To evaluate the elicitation of neutralising antibodies against the yeast expressed Hemagglutinin recombinant protein, BALB/c mice and rabbits were immunised intramuscularly after mixing with Freund's complete adjuvant. An average antibody titre of 1:512 was observed in case of mice group immunised with 50μg of HA after two weeks of immunization, whereas, the mice group that received 10μg of HA had an antibody titre of 1:256. Interestingly both the immunised rabbits showed better seroconversion as early as two weeks of immunization with an antibody titre of 1:2048 when compared to the control non immunised animal. Following two weeks of booster, the antibody titres shot up to 1:2048 and 1:1024 in case of mice group immunised with 50 μg and 10 μg of HA respectively. Both the immunised rabbits had an antibody titre of 1:8192 (table 1) after two weeks of booster injection.

**Table 1:** Concise summary of the Immunization study of yeast derived HA protein in mice and rabbits

Animal group	Sera sample	Antibody titre	HI titre^#^	PRNT titre^#^
Mice group V1 (50μg/dose/animal)	Pre Immune	**<1:16**	**0**	**<16**
	
	2 wks post Immunisation	**1:512**	**32**	NT*
	
	2 wks post booster	**1:2048**	**32**	**256**

Mice group V2 (10μg/dose/animal)	Pre Immune	**<1:16**	**0**	**<16**
	
	2 wks post Immunisation	**1:256**	**32**	NT*
	
	2 wks post booster	**1:1024**	**32**	**64**

Mice control group	Pre Immune	**<1:16**	**0**	**<16**
	
	2 wks post Immunisation	**<1:16**	**0**	NT*
	
	2 wks post booster	**<1:16**	**0**	**<16**

Rabbit 1 (50μg/dose/animal)	Pre Immune	**<1:16**	**0**	**<16**
	
	2 wks post Immunisation	**1:2048**	**32**	NT*
	
	2 wks post booster	**1:8192**	**64**	**256**

Rabbit 2 (50μg/dose/animal)	Pre Immune	**<1:16**	**0**	**<16**
	
	2 wks post Immunisation	**1:2048**	**32**	NT*
	
	2 wks post booster	**1:8192**	**64**	**256**

Rabbit Control	Pre Immune	**<1:16**	**0**	**<16**
	
	2 wks post Immunisation	**<1:16**	**0**	NT*
	
	2 wks post booster	**<1:16**	**0**	**<16**

NT* - Not tested; # HI and PRNT titres are the mean reciprocals obtained against the H1N1 virus isolated during the 2009 outbreak in India

### Hemagglutination Inhibition (HI) activity of H1N1HA immunised mice and rabbit sera

**Figure 6:**
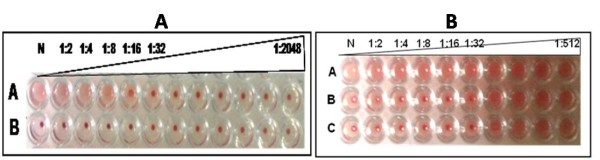
**Hemagglutination (HA) activity of H1N1 virus and Hemagglutination Inhibition (HI) activity of HA immunized sera using chicken RBCs**.Panel 6A: Hemagglutination (HA) activity of H1N1 virus. Lane A: Two fold serial diluted MDCK derived H1N1 virus mixed with 0.5% chicken RBCs. Lane B: Negative control (PBS mixed with 0.5% chicken RBCs). Panel 6B: Hemagglutination Inhibition (HI) activity of HA immunized sera using chicken RBCs. Lane A: Negative control (None immunized mice serum mixed with 4HA units of H1N1) Lane B: Two fold serially diluted HA immunized representative mice serum mixed with 4HA units of H1N1 Lane C: Two fold serially diluted HA immunized representative rabbit serum mixed with 4HA units of H1N1.

The HI assay is the most widely accepted serological test for influenza immunity and is the gold standard measure of functional HA-specific antibodies after vaccination. The MDCK derived H1N1 virus agglutinated chicken RBCs up to 1:8 dilutions and hence this was considered as 1HA unit (Figure 6A). After two weeks of initial immunization, HI titres were induced in both immunised mice groups and rabbits with mean HI titres reaching about 1:32 (Figure 6B). After two weeks of booster injection, the HI titres remained same in both immunised mice groups. Whereas the rabbits showed an elevated titres of 1:64 (table 1).

### *Invitro *neutralisation activity of H1N1HA immunised mice and rabbit sera (Plaque Reduction Neutralisation Test)

**Figure 7:**
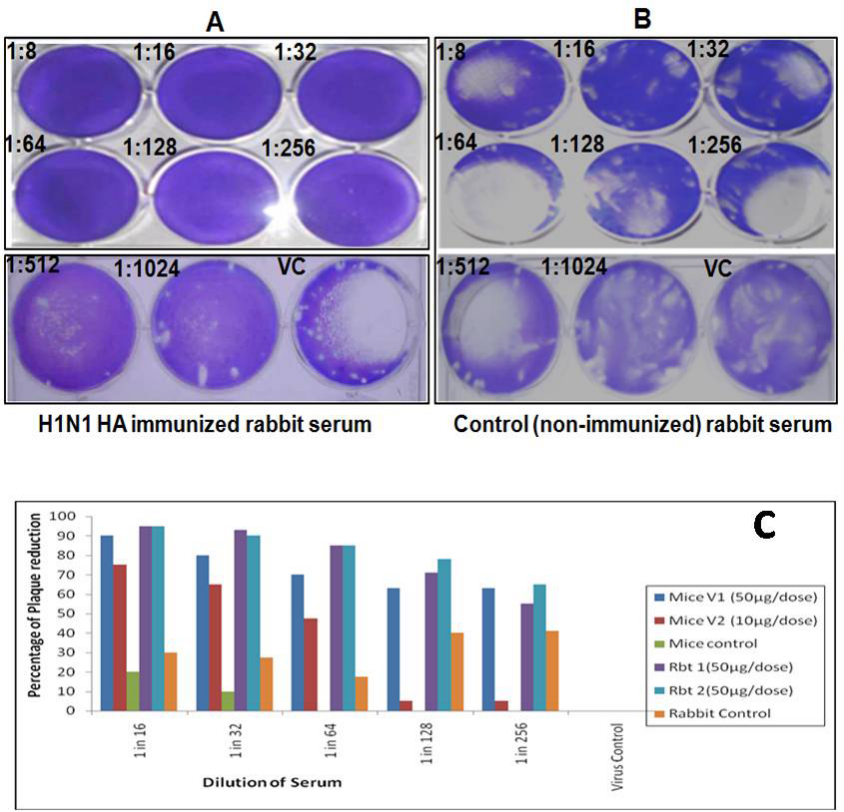
**Plaque Reduction Neutralization Test (PRNT) of HA immunized sera demonstrating virus neutralization activity against H1N1 virus**. Panel 7A and 7B: Representative immunized rabbit serum showing virus neutralisation activity against H1N1 virus (panel A) compared to the non immunised control rabbit serum (panel B) Panel 7C:Graph depicting the PRNT titres (50% plaque reduction) of immune mice/rabbit sera samples in comparison to non immune sera samples.

Plaque Reduction Neutralisation Test (PRNT) was performed on the sera samples collected after two weeks of booster immunisation both in case of mice and rabbits. The serially diluted sera were challenged with 100 pfu showed 50% reduction in plaque numbers up to 1:256 dilutions in case of mice group that received 50μg of HA protein. However, the mice group that were immunised with 10μg of HA protein had more than 50% reduction in plaque numbers only up to 1:64 dilution. The immunised rabbit sera also showed a neutralizing titres of 1:256 up to which there was 50% reduction in the plaque numbers (Figure 7, panel A). Whereas, the sera from the control mice group and rabbit did not show any significant reduction in the plaque numbers at any of the dilutions tested (Figure 7, panel B). Figure 7 (panel C) depicts the different PRNT titres obtained with each animal group.

## Discussion

*Pichia pastoris *has been used successfully to express a wide range of heterologous proteins [12,24,26,32-36,41,42]. Heterologous expression in *P. pastoris *can be either intracellular or secreted. The major advantage of expressing heterologous proteins as secreted protein is that *Pichia pastoris *secretes very low levels of native proteins. That combined with the very low amount of protein in the minimal *Pichia *growth medium, means that the secreted heterologous protein comprises the vast majority of the total protein in the medium and serves as the first step in purification of the protein [43]. In the present study we have generated recombinant Pichia clones in which multiple copies of H1N1 HA transgene are integrated. The recombinant HA protein is also separated from secretory signal by the action of host specific endopeptidase resulting in the release of the matured, fully processed HA protein. Unlike bacteria the transgene in case of yeast is integrated within the genome, hence it is difficult to lose the target gene when the recombinant yeast is cultured and passaged several times. A further advantage of selecting for multi-copy transformants is that if there is a mutation in one particular copy of the expression cassette, arising from the integration process, then the protein that results from this mutant copy may not contribute as significantly to the total amount of protein expressed. In case of native Influenza virus, the monomers of HA folds to form a membrane proximal stalk and a membrane distal globular head domain. The globular head stands independently from the central stalk and contains the majority of the neutralizing antibody epitopes and the HA monomers are oligomerised into HA trimers [44,45]. Hence in order to have a neutralising immune response from protein based vaccine, HA in its native form may be the best target. The full length and partial H1N1 HA protein has been previously expressed in bacterial system [46]. There is a report on lower level of expression of Human Influenza A/WSN/33 HA protein in *S. cerevisiae *[47]. However this reported protein is truncated, hyperglycosylated and is also cell-associated and have not demonstrated its *in vivo *applications. Xavier Saelens *et al *have successfully expressed the H3N2 HA protein in *Pichia pastoris *as a soluble secretory protein in its monomeric form but not as trimeric form [48]. However they have also have demonstrated protective efficacy of the monomeric H3N2 HA in mice model. But there are no reports on the expression of pandemic H1N1 viral protein using *Pichia pastoris *expression host so far. Here we demonstrate that that *P.pastoris *is capable of expressing a soluble form of H1N1 HA with near native antigenic structure in trimeric form. Furthermore, the yeast derived recombinant HA is also capable of eliciting a good neutralising antibody response in mice and rabbits.

From the current study it is evident that in order to have a good expression of full length H1N1 HA gene, multiple copy integrants carrying more than 4 copies of the target gene are essential. The mixture of trimers and monomers observed in the present study could be attributed to the influence of extracellular pH. This is in accordance with the previous report wherein the extra cellular pH has influenced the trimerization and transport of HA from the mammalian host cells [49]. Even though the pH of the induction medium was initially adjusted to 8.2, at the time of protein harvest, drop in pH to acidic range (pH 6-7) was noticed which probably would play a role in inhibiting the oligomerization of the secreted HA monomers. As it was shaker flask culture, it was practically difficult to continuously monitor the extra-cellular pH and in addition it seems unlikely that if there is any trimerization of HA happening within the yeast cell, the aggregated HA trimers of high molecular weight could traverse the yeast cell wall. In order to have correctly folded glycosylated protein, full length HA synthetic gene was used in the present study for expression in yeast. Out of several clones screened, two clones carrying multiple copies of HA gene showed better expression of the target protein, whereas the other clones had weak expression of the target protein. This observed reduced expression level may be attributed to the low copy number of the transgene integrated within the yeast genome. This is in agreement with the earlier reports wherein multi-copy recombinants or the jackpot clones increases the expression levels of the target protein due to more number of copies or gene dosage [31,33]. SDS-PAGE and Western blotting analysis revealed the expression of a specific protein, whose molecular weight was approximately 80 KDa from recombinant *P. pastoris *and this expressed protein reacted with H1N1 HA specific antibodies. The Monomers of the expressed HA also formed trimers similar to the HA trimers on the native virus. The trimeric HA was of higher molecular weight of approximately 240 KDa as seen on the native PAGE. These trimeric HA proteins under denaturing conditions, got dissociated into its constituent monomers. The H1N1 HA specific antibodies used in the present study also have confirmed the authenticity of the HA trimers. It is noteworthy to mention that the HA gene expression was noticed up to five passages during the course of this study indicating good genetic stability of the introduced HA gene within the recombinant yeast system.

During optimisation, all though different harvest time points were tested, the protein bands were clearly visible only in case of the samples that were incubated for 46 hr of post methanol induction and band intensities were significantly reduced in the time points collected before 46 hr of post induction. Prolonged incubation after 46 hrs up to 96 hrs post induction resulted in the breakdown of the target protein. Very faint but multiple bands were observed following 72 hr and no bands were visible after 96 hr of incubation indicating that host-specific proteases may be acting on the protein following prolonged incubation. These findings clearly suggest that early harvesting of the culture supernatant is necessary to have an intact H1N1 HA protein without any proteolytic damage. In addition to the earlier discussion on the influence of pH for obtaining HA trimers, pH of the medium also played a critical role in the stability and integrity of the HA protein expressed extra-cellulary. It was observed that the acidic pH is not suitable for the stability of the expressed protein. Rather slightly alkaline pH (8.2) showed very good stability of the expressed recombinant HA protein. As Pichia cells are better adapted for growth under acidic environment, the increased pH of the medium had some adverse effects on the biomass. The culture was also harvested at an early stage (46 hrs), this would be another reasons for not getting a high cell density using shaker flask culture. Another important parameter for efficient expression of HA in Pichia was adequate aeration during methanol induction. Hence the culture volume of the flask was kept as low as 10% of the total flask volume. It was also necessary to maintain the incubation temperature at 28°C with rotation of 250 rpm. Under optimal condition, the expressed protein amounted to be about 60 mg/Lt of the culture in shaker flask condition. As this yield obtained was under normal shaker culture conditions, it should be possible to obtain several fold higher expression levels by optimized fermentation procedures, allowing cell densities of A600 = 200 ± 400 instead of 5 ±10.

Yeast expression system like *S. cerevisiae can *hyper glycosylate the recombinant protein with high mannose-type oligosaccharides and hence the recombinant protein can be recognized by mannose receptors when injected into mammalian species [50-52]. *S. cerevisiae *glycosylation has terminal α-1, 3 linked mannose residues and it is this residue that is thought to be antigenic. Whereas *P. pastoris *does not have this terminal link [50,53] and hence is a better system for recombinant antigen expression. H1N1 HA derived from yeast in the present study was found to be less immunogenic but efficacious in eliciting good neutralising immune response in mouse model. From the Coomassie stained gels, it is evident that majority of the protein secreted into the medium was the HA protein with negligible amount of non specific host proteins. Hence this study is very significant for easy and economical downstream processing of the recombinant protein.

From the preliminary immunisation experiments conducted, it is evident that mice receiving the recombinant HA as low as 10μg were able to induce a neutralising antibody titre of 1:64. Whereas both mice and rabbits that received 50μg of recombinant protein had a neutralising antibody titre of upto 1:256. However the present studies in animal models are in proof of concept stage wherein the yeast derived recombinant H1N1 HA protein has been demonstrated to elicit virus neutralizing antibodies. Appropriately designed biological challenge experiments with various concentrations of the recombinant protein in combination with different types of adjuvants and comparative studies with the commercially available vaccines are in progress. Previous studies [8] have demonstrated micro-neutralisation titres of 1:160 in ferrets. Thus the micro neutralisation titre obtained in the present study is quite encouraging and probably sufficient enough to provide complete protection against the lethal virus challenge.

The H1N1 Hemagglutinin protein is known to carry neutralizing epitopes, hence the yeast derived glycosylated HA protein obtained from our study may find dual applications in both disease diagnostics and prophylaxis. As the main objective of the present study was confined to the recombinant expression of H1N1 HA using *P.pastoris*, detailed studies pertaining to the prophylaxis and diagnostic potential of this recombinant protein were beyond the scope of this study at this stage. However in another study we have initiated evaluating the diagnostic potential of the yeast derived HA protein either in detecting H1N1 specific antibodies in human serum samples or in direct detection of H1N1 virus in clinical samples employing chicken polyclonal antibodies raised against the recombinant HA protein. The key benefits of using yeast for expressing H1N1 HA protein are that the recombinant protein can be made quickly, inexpensively and in quantities sufficient to meet global needs. The efficiency of this technology translates several fold increase in production. As a point of reference, the average yield for cell culture is 3 mg/Lt; for egg based production, 7 mg/Lt; for baculovirus recombinant synthetic protein, 13 mg/Lt [45]. Whereas for the standard yeast system described here we could able to reach an yield of 60 mg/L using shaker flask culture and this yield can be certainly elevated further using fed batch fermentation. This increase in production capacity, along with the fact that it is carried out in a yeast system, provides an opportunity to address several shortcomings of the current egg-based system. One advantage deriving from increased capacity is the ability to increase the dose of antigen. Formulation of a ''high-dose'' vaccine for the elderly becomes a practical possibility with an unconstrained supply of antigen. A second set of advantages comes from eliminating the growth of virus from the manufacturing process.

## Conclusion

We have successfully expressed the trimeric hemagglutinin protein of H1N1 using yeast system (*Pichia pastoris*) in secreted form. The yeast derived HA protein is capable of eliciting virus neutralising antibodies in both mice and rabbit models. The sera from immunised animals also exhibited Hemagglutination Inhibition (HI) activity. Hence *Pichia pastoris *may be considered as an appropriate alternate for the development of an easily adaptable, safe and economic alternative HA based subunit vaccine. Although the mouse model used here is commonly accepted to evaluate experimental influenza vaccines, the results described should only be regarded as initial proof of principle. The manufacturing approach described here can be further scaled up to high levels of productivity and the flexibility and potential speed associated with yeast expression system may prove to be indispensable during pandemic influenza outbreak.

## Materials and methods

### Yeast strain and growth conditions

*P. pastoris GS115 *(Invitrogen, USA) was grown at 28°C in YPD medium (Yeast Extract Peptone Dextrose Medium). For growth on plates, 2% agar was added to the media. Transformants were grown in media supplemented with 250-750 μg/ml Geneticin (Sigma, USA). For cloning procedures, *Escherichia coli DH5 α *were used and grown at 37°C in LB medium supplemented with 50 μg/ml either kanamycin or ampicillin.

### DNA techniques

Molecular biology protocols were carried out according to Sambrook and Russell [21]. *E. coli *and *P. pastoris *cells were transformed by Electroporation. Enzymes *Eco *RI, *Not *I, *Sal *I and T4 DNA ligase (Fermentas, USA), *Taq *DNA polymerase and reagents for PCR (Invitrogen, USA) were used as recommended by the supplier. DNA sequencing was performed by ABI DNA sequencer (Applied Biosystems, USA). DNA sequences were analyzed using DNA star software.

### Cloning H1N1 HA gene into pPICK9K yeast transfer vector

The DNA corresponding to nucleic acids 1 to 1699 of the HA gene from novel California/04/2009 H1N1 [Genbank:FJ966082.1] was synthesized by Biotech desk (Hyderabad, India). The full length HA-encoding synthetic gene was PCR amplified from the synthetic construct using high fidelity *Pfu *Taq polymerase (Fermentas, USA) employing the following primers having introduced *Eco *RI and *Not *I sites in the forward and reverse primers respectively. H1N1HA Forward: 5'- TTG GAT CCA **GAA TTC **ATG AAG GCA ATA CTA GTA GTT CTG-3' [Base pairs: +1 to +24 Genbank: FJ966082.1]; H1N1HA reverse: 5'-TGG ATC C**GC GGC CGC **AAT ACA TAT TCT ACA CTG TAG AGA -3' (Base pairs: +1699 to +1681 NCBI Genbank: FJ966082.1]. The PCR conditions used were: 94°C for 45 sec, 63°C for 45 sec, 72°C for 1 min and 30 sec, for 35 cycles, and finally 72°C for 10 min. The amplified HA gene was digested with *Eco *RI and *Not *I restriction enzymes and was cloned into pPIC9K yeast transfer vector (Invitrogen, USA) at the same restriction sites. The resulting vector pPICK9KH1N1HA (Figure 1) had the HA gene in frame with the fused *Saccharomyces cerevisiae *α-mating factor secretion signal under control of the methanol-inducible *P. pastoris *alcohol oxidase 1 (AOX1) promoter. [The complete sequence of the resultant pPIK9KH1N1HA recombinant construct is submitted to NCBI Genbank: HQ398363.1]. The pPICK9KH1N1HA DNA was transformed into *E.coli *DH5 alpha strain (Invitrogen, USA) *via *heat shock method. For selection of the recombinant transformants, the bacterial cells were cultured in Luria-Bertani medium (Himedia, India) supplemented with 50μg/ml ampicillin and 50 ug/ml of Kanamycin. The positive bacterial transformants were selected through restriction digestion of plasmid DNA using *Eco *RI and *Not *I enzymes and PCR analysis using alpha factor forward (5'- TAC TAT TGC CAG CAT TGC TGC-3') and H1N1 HA reverse primers. Correct integration will result in the formation of a 1.96 Kbp PCR product. The complete HA gene sequence was further confirmed through nucleotide sequencing using ABI sequencer for any possible mutations introduced during PCR step.

### Integration of pPICK9KH1N1HA DNA into *Pichia pastoris *genome and screening of the transformants

The recombinant plasmid DNA pPIC9KH1N1HA was linearized by digesting with *Sal *I enzyme to integrate the transgene at *His*4 locus on the Pichia genome and also to generate HIS+, Mut+ transformants in *Pichia pastoris *GS115 cells. Ten microgram of the linier DNA was used to transform fresh electro competent *P. pastoris *cells *via *electroporation using Bio-Rad Gene Pulsar Xcell™ electroporation system (Bio-Rad laboratories, Inc USA.) at three different voltages (1600V, 1800V and 2000V), 20μF capacitance and 200Ω resistance. After transformation, cells were plated on SD-His plates (1.34% yeast nitrogen base, 2% dextrose, 0.01% complete amino acid supplement minus Histidine, 1 M sorbitol supplement, and 2% agar), and incubated at 30°C for 2 days. The parent pPIC9K without insert, linearized with *Sal *I was also transformed similarly for negative control. The colonies obtained were streaked on fresh SD-His plates. It is often desirable to select for transformants containing multiple integration events (Figure 1B) as such clones potentially express significantly higher levels of the recombinant protein. Three hundred and sixty five transformed colonies bearing the chromosomally integrated copies of the pPICK9KH1N1HA were screened for single, double or multiple copy integrants through replica plating on YPD plates containing different concentrations of Geneticin (250μg, 500μg and 750μg). Plates were incubated at 30°C for four days and the growth obtained was scored with plus (+) and minus (-) for the presence or absence of growth respectively on the selection plate. Since both Kan^R ^and H1N1HA gene are integrated together, resistance to Geneticin would indicate the copy number of the integrated H1N1 HA gene. As a reference, clones that grow well on selection plate with Geneticin concentration of 250μg/ml, 500μg/ml and 750μg/ml were considered to have single, double and more than four copies integrated respectively. To further confirm the transformants having multiple copy integrants, PCR was performed on the genomic DNA isolated from selected colonies by employing alpha factor secretary signal forward and H1N1HA reverse primers. A total of twenty clones were randomly picked up from all the three categories (3, 6 and 11 clones each from 250μg/ml, 500μg/ml and 750μg/ml plates respectively) and were inoculated into 10 ml of YPD broth and grown at 28°C until the OD was 2-3. The OD in all was finally adjusted to 2 using the blank YPD medium and 10 ml of each culture was further used for Genomic DNA extraction. The template DNA concentrations in all cases were equally adjusted in the PCR reactions to check its sensitivity to screen single, double and multiple copy integrants along with appropriate control as reported earlier [41]. Fourteen PCR positive Pichia clones that were found to contain single, double and more than four copies of the H1N1HA gene integrated were selected further for subsequent expression studies.

### Expression analysis and optimization of H1N1 HA protein expression in Pichia system

Fourteen PCR positive His+ Mut+ Pichia clones were selected for methanol induction. The glycerol stocks of the above Pichia clones were inoculated separately into 50-ml of either YPGy (1%Yeast extract, 2%bacto peptone and 1%glycerol buffered with 100 mM potassium phosphate buffer, pH 8.2) or Buffered Minimal Glycerol medium-BMGM (100 mM potassium phosphate, pH 8.2, 1.34% YNB, 4 × 10^-5 ^% biotin, 1% Glycerol) taken in 500 ml conical flask along with negative control (Pichia transformed with pPICK9K without insert) and were incubated at 28°C in a shaker incubator at 250 rpm until the culture reached an A600 of 4-5. The cells were harvested by centrifugation at 3,000 × g for 10 min at room temperature and the cell pellets were resuspended in required volume of either fresh YPM induction media (1%Yeast extract, 2%bacto peptone and 0.5 - 2.5% Methanol, buffered with 100 mM potassium phosphate buffer, pH 8.2) or Buffered Minimal Methanol medium-BMM (100 mM potassium phosphate, pH 8.2, 1.34% YNB, 4 × 10^-5 ^% biotin, 0.5%- 2.5% methanol) so as to get an A600 of 3 in all. Several methanol concentrations ranging from 0.5 to 2.5% (0.5%, 1%, 1.5%, 2% and 2.5%) were tried in both media in order to choose the optimum concentration of methanol for induction in case of shaker flask culture. Incubation was continued at 29°C on an orbitary shaker (250 rpm) for four days. To sustain induction, required volume of methanol was added to every flask once in every 24 hour. Culture supernatants were collected at different time points ranging from 16-96 h (16 h, 23 h, 46 h, 72 h and 96 h) and were concentrated to 1/10 of its original volume using cellulose membrane with a pore diameter of 10 KDa (Millipore Corporation, USA) by centrifuging at 4000 g for 10-20 min at 4°C. Protease inhibitor cocktail (Amersco, USA) was added to the concentrated samples and the samples were stored at -80°C until all the time points were collected. Two clones with multiple copy integrants (Showing Geneticin resistance up to 750μg/ml and also resulted in intensive HA amplified PCR product) showing high expression of a recombinant protein were further selected for subsequent optimization experiments. The expression conditions *viz *methanol concentration (0.5-2.5%), type of medium (YPGy or BMGM), pH of the medium (6 - 8.5) and the time of harvest (16 h, 23 h, 46 h, 72 h and 96 h) were tested in order to get the intact HA protein expressed in abundance without any host specific proteolytic cleavage. The final protein obtained after optimization of the expression conditions obtained from cell culture supernatants were determined through Bradford assay against BSA standards [54]. The protein samples were further analyzed by running them on 10% polyacrylamide gel electrophoresis (PAGE) both under denaturing and native conditions [21]. The gels were subsequently stained with Coomassie Brilliant Blue R-250 (Sigma, USA). The expressed HA protein was confirmed through western blotting using rabbit anti H1N1 HA specific polyclonal antibodies (Genscript, USA) and goat anti rabbit alkaline phosphatase conjugated IgG (Sigma, USA) as primary and secondary antibodies respectively. The colour development was done either using BCIP/NBT solution (Sigma,USA) or H2O2/DAB substrate/chromogen (Sigma,USA).

### Concentration of HA protein

The culture supernatants showing high level of secreted expression under optimal expression conditions were collected after methanol induction. The protein were concentrated ten times using 10 KD MWCO spin columns (Millipore,USA) by centrifuging at 4000 g for 20-30 min at 4°C.

### FPLC purification of the Yeast derived HA protein based on size exclusion principle

The Yeast expressed protein recovered from the yeast culture supernatant was subjected to fast protein liquid chromatography (FPLC) using Akta explorer (Amersham, USA) employing previously equilibrated (in two column volumes of 20 mM Phosphate Buffered Saline,pH 7.2) Superdex 200 10/300 column (GE-Healthcare, USA). The Hemagglutinin protein of concentration 3 mg/ml diluted in 2.5 ml of Phosphate Buffered Saline (pH 7.0) was injected into the size exclusion chromatography column and the proteins with different sizes were eluted by monitoring the protein at 280 nm. The peaks obtained were compared with the known molecular weight marker proteins (GE-Healthcare, USA). Fractions corresponding to monomers and trimers of HA were collected separately for further use.

### Analysis of the recombinant HA protein for trypsin cleavage

The yeast derived HA protein was checked for its cleavage into HA1 and HA2 fragments *via *trypsin digestion. To 10μg of yeast expressed HA protein, trypsin (100μg/ml stock made in PBS, pH7.2) was added to a final concentration of 1μg/ml and incubated at 37°C for one hour. The digested HA protein sample along with the negative control (HA protein undigested with trypsin) were run on 10% native PAGE and stained with coomassie brilliant blue.

### Immunization of mice and rabbits with yeast derived H1N1HA recombinant protein and detection of serum antibodies through antibody capture ELISA

The animal studies experiments had an approval from the Institutional Animal Ethics Committee (IAEC) wide registration number 37/1999/CPCSEA and Institutional Biosafety committee (IBSC) wide reference no: IBSC/VIRO-01/05/TNA as per the institutional norms. The principles of good laboratory animal care were followed all through the experimental process. Eighteen healthy BALB/c mice 6-8 week-old were made into three groups with six animals in each group. The animals were found to be sero negative for the circulating H1N1 influenza. Two groups were immunized intramuscularly each with 50μg and 10μg of yeast derived H1N1 HA protein in Freund's complete adjuvant (FCA) (Sigma,USA) and the animals of the control group were immunized with the expressed product of negative control *P. pastoris*. Similarly two healthy adult male New Zealand White rabbits tested sero negative for H1N1 were immunized intramuscularly with 50μg each of yeast derived HA protein in combination with FCA for negative control, one rabbit was similarly immunized with FCA alone. After two weeks, animals were boosted once with the same amount of protein in combination with Freund's Incomplete adjuvant (FIA) (Sigma, USA). Following two weeks of the booster dose, blood samples were collected either from retro-orbital route (In case of mice) or marginal veins (rabbits) and the sera were separated. Antibody capture ELISA was performed to determine the antibody titre against the H1N1HA protein [16]. Briefly, the yeast expressed H1N1 HA protein was coated overnight onto Nunc polystyrene microtitre plates (300 ng/well in 0.1 M sodium bicarbonate, pH 8.0) and the non reacted sites were blocked using 3% BSA. The captured proteins were reacted first with the immunised mice and rabbit sera and were subsequently incubated with their respective anti-species secondary HRP conjugates (Sigma, USA) for 1 hour. Enzymatic colour development was done using TMB/H2O2 chromogen/substrate solution. The samples showing the OD values twice that of negative serum were considered to positive and were used in determining the titre of the antibody.

### Culturing H1N1 virus and determination of its Hemagglutination (HA) titre and virus quantification through Plaque assay

The H1N1 virus isolate from clinical sample in Bangalore, India during 2009 outbreak was a kind gift from Dr.V.Ravi, NIMHANS, Bangalore, India. The H1N1 virus was propagated using Madin Darby Canine Kidney (MDCK) cell lines as described earlier [45,49]. Hemagglutination assay was performed on the H1N1 virus stock using chicken RBC. Briefly, chicken RBCs were separated from the whole blood and washed three times in PBS (pH7.2). Fifty micro litres of 0.5% RBC suspension (v/v in 1% PBS) was added to 50μl serially diluted H1N1 influenza virus in U-bottom 96 well plates. The plates were incubated at room temperature for one hour and were observed for the formation of button or mat within the wells.

For quantifying the H1N1 virus, plaque assay was performed by serially diluting the virus stock on MDCK cells in six well plates. The plaques formed after three days of post infection were counted and the titre of the virus stock was determined. The H1N1 virus stock with known HA titre and virus concentration was used for subsequent experiments. The culturing of the virus, determination of Hemagglutination (HA) test and plaque assay were performed in Bio safety level 3 (BSL-3) laboratory.

### Hemagglutination Inhibition (HI) activity of H1N1HA immunised mice and rabbit sera

Two fold dilutions of immunised/control mice and rabbit sera were made in U-bottom 96-well micro titre plate. Four Hemagglutination units (HAU) of influenza virus were added in each well and the virus-serum mixture was incubated for 30 minutes and 0.5% suspension of chicken RBC (in PBS pH7.2) were added and mixed by agitation. The chicken RBCs were allowed to settle for one hour at room temperature and HI titres were determined by the reciprocal value of the last dilution of the sera which completely inhibited the Hemagglutination of chicken RBCs.

### *In-vitro *neutralisation activity of H1N1HA immunised mice and rabbit sera

Serum samples were heat-inactivated at 56°C for 30 minutes. Two-fold serial dilutions from 1:16 to 1:1024 were prepared in virus diluent (MEM with L-glutamine) containing no serum or antibiotic/antimycotic solution. Serially diluted serum was challenged with an equal volume of the H1N1 virus, previously titrated to give 100 pfu in 250 μl of virus dilution. The virus control of the experiment contained the virus diluted in the virus diluents without serum. The serum/virus mixtures were incubated at 37°C, 5% CO_2 _for one hour. MDCK cell monolayers, prepared in six well plates were infected with 500 μl/well of the serum/virus mixture. Plates were incubated at room temperature for one hour. The supernatants were completely aspirated out from the wells and the wells were overlaid with 1.5% low melting point agarose (Sigma,USA) prepared in 2X MEM containing 5 μg/ml trypsin. Plates were incubated for plaque formation at 37°C, 5% CO_2 _for 3 days and the wells were stained with 0.2% crystal violet solution (made in 30% ethyl alcohol). The plaques formed were counted and neutralisation activity of the immune sera was assessed by comparing the plaque numbers obtained from that of negative control serum. The highest dilution of the sera that showed more than 50% reduction in plaque number than that of negative control is considered as the neutralising titre.

## Competing interests

The authors declare that they have no competing interests.

## Authors' contributions

Conceived and designed the experiments: TNA, Performed the Experiments: TNA, SS, SRS, AKS, RP; Analysed the data: TNA, PVL, NG, MMP, RV, VVS; Contributed reagents/materials/analysis tools: VVS; Wrote paper: TNA. All authors have read and approved this manuscript.

## Author's information

Athmaram TN is working as Scientist at Division of Virology, DRDE, Gwalior, India and has vast experience on viral vaccines from different heterologous expression systems like yeast, bacteria, baculovirus and mammalian systems from past ten years. SS, AKS, RP are Research fellows at DRDE, Gwalior, PVL, NG, MMP, RV and VVS have expertise in the area of Virology and recombinant DNA technology.
